# aP2 Protein Expression as a Diagnostic Marker in Soft Tissue Tumours

**DOI:** 10.1080/13577140120048593

**Published:** 2001-09

**Authors:** T. T. Yang, A. C. C. Reed, N. A. Athanasou

**Affiliations:** 1 Department of Pathology Nuffield Department of Orthopaedic Surgery University of Oxford Nuffield Orthopaedic Centre Oxford OX3 7LD UK

## Abstract

*Purpose/Methods:* The aP2 gene product (aP2 protein) is known to be expressed by preadipocytes and other immature fat cells in vitro. A mouse monoclonal antibody raised against an 18 amino acid segment of the aP2 protein was found to react with lipoblasts and fetal fat cells in paraffin sections of soft tissue tumours of adipose differentiation. In this immunohistochemical study, we have further examined the diagnostic utility of aP2 expression in distinguishing tumours of adipose differentiation from other benign and malignant soft tissue tumours.

*Result and discussion* aP2 was strongly expressed by lipoblasts in lipoblastomas and all types of liposarcoma as well as brown fat cells in hibernomas. Optimal conditions for immunohistochemical identification of lipoblasts in tumours of adipose differentiation was noted when the antibody was diluted 1:30 to 1:50. Small lipoblast-like fat cells in pleomorphic lipoma and spindle cell lipoma also showed variable staining for aP2 at this dilution of the antibody. Most benign and malignant soft tissue tumours were distinguished by their absence of staining for aP2 protein, but some cases of myxoma, malignant fibrous histiocytoma, synovial sarcoma and leiomyosarcoma contained tumour cells which reacted for aP2. aP2 protein expression is likely to prove a useful means of distinguishing lipoblasts in liposarcoma but it should be used as part of a tumour panel to exclude expression in other forms of mesenchymal tumour.


*Sarcoma* (2001) 5, 139–142

ORIGINAL ARTICLE

### aP2 protein expression as a diagnostic marker in soft tissue tumours

T.T. YANG, A.C.C. REED & N.A. ATHANASOU*

**Department of Pathology, Nuffield Department of Orthopaedic Surgery, University of Oxford, Nuffield Orthopaedic Centre, Oxford OX3 7LD*

**Summary**

*Purpose/Methods:* The aP2 gene product (aP2 protein) is known to be expressed by preadipocytes and other immature fat cells in vitro. A mouse monoclonal antibody raised against an 18 amino acid segment of the aP2 protein was found to react with lipoblasts and fetal fat cells in paraffin sections of soft tissue tumours of adipose differentiation. In this immunohistochemical study, we have further examined the diagnostic utility of aP2 expression in distinguishing tumours of adipose differentiation from other benign and malignant soft tissue tumours.

*Result and discussion* aP2 was strongly expressed by lipoblasts in lipoblastomas and all types of liposarcoma as well as brown fat cells in hibernomas. Optimal conditions for immunohistochemical identification of lipoblasts in tumours of adipose differentiation was noted when the antibody was diluted 1:30 to 1:50. Small lipoblast-like fat cells in pleomorphic lipoma and spindle cell lipoma also showed variable staining for aP2 at this dilution of the antibody. Most benign and malignant soft tissue tumours were distinguished by their absence of staining for aP2 protein, but some cases of myxoma, malignant fibrous histiocytoma, synovial sarcoma and leiomyosarcoma contained tumour cells which reacted for aP2. aP2 protein expression is likely to prove a useful means of distinguishing lipoblasts in liposarcoma but it should be used as part of a tumour panel to exclude expression in other forms of mesenchymal tumour.

**Keys Words:** *adipose tissue, lipoblasts, liposarcoma, lipoma*

### Introduction

Benign and malignant tumours of adipose differentiation are amongst the most common of soft tissue tumours.[1] Morphological identification of atypical lipoblasts is essential for the diagnosis of liposarcoma as these cells are not present in other types of malignant soft tissue tumour. With the exception of lipoblastoma, and to a lesser extent pleomorphic lipoma and spindle cell lipoma, lipoblasts are absent from benign tumours of adipose differentiation. Morphological identification of lipoblasts may be problematic, however, as these cells can be few in number or difficult to identify in well-differentiated liposarcomas. They may be also difficult to distinguish morphologically from vacuolated tumour cells in non-adipose benign and malignant mesenchymal soft tissue tumours.

We have previously shown that both polyclonal and monoclonal antibodies raised against the aP2 protein can be used to identify lipoblasts and fetal fat cells in tumours of adipose differentiation immunohistochemically.[2,3] aP2 expression was noted in liposarcomas, lipoblastomas and hibernomas. The aP2 gene (also known as P422 and adipocyte lipid binding protein) codes for a 132 amino acid, single chain 14.6kd polypeptide which shows considerable homology with myelin P2 protein.[4,5] aP2 expression is coincident with acquisition of the morphological features of the adipocyte phenotype [4] and aP2 is expressed in preadipocytes late in adipogenesis.[6]

In this study, we have determined whether the aP2 protein is expressed in a range of benign and malignant soft tissue tumours, including various types of tumours of adipose differentiation. We have also sought to characterise the optimal conditions required for immunohistochemical staining using the monoclonal antibody which identifies this antigen. On this basis, we have sought to determine whether aP2 expression can reliably be used as a marker of lipoblasts and fetal fat cells in the diagnosis of soft tissue lesions.

### Materials and methods

The anti aP2 antibody used in this study is a non-commercial monoclonal antibody raised against a synthetic peptide corresponding to the 18 amino acid sequence of residues 10–28 of the aP2 protein.[3] The antibody was not affinity purified. 5μm paraffin sections of

*Author/address for correspondence: N. A. Athanasou, Department of Pathology, Nuffield Department of Orthopaedic Surgery, University of Oxford, Nuffield Orthopaedic Centre, Oxford OX3 7LD. Fax: 01865 742348; Tel: 01865 227619; E-mail nick.athanasou@ndos.ox.ac.uk

1357–714X print/1369–1643 online/01/030139–04 © 2001 Taylor & Francis Ltd
DOI: 10.1080/13577140120071678

**Table 1.** *aP2 expression in soft tissue tumours*

| Soft tissue tumours | Number of cases of each tumour studied | Number of cases showing aP2 reaction (dilution 1:30) |
|---|---|---|
| *Tumours of adipose differentiation* | | |
| Lipoma | 12 | 0 |
| Pleomorphic lipoma | 3 | 1 |
| Intramuscular lipoma | 4 | 0 |
| Spindle cell lipoma | 6 | 2 |
| Lipoblastoma | 3 | 3 |
| Hibernoma | 3 | 3 |
| Liposarcoma | | |
| well differentiated | 13 | 11 |
| myxoid | 8 | 6 |
| round cell | 5 | 5 |
| pleomorphic | 6 | 5 |
| *Other mesenchymal tumours* | | |
| Myxoma (intramuscular) | 6 | 1 |
| Benign fibrous histiocytoma | 2 | 0 |
| Fibromatosis (extra-abdominal) | 5 | 0 |
| Glomus tumour | 2 | 1 |
| Haemangioma | 2 | 0 |
| Neurilemmoma | 2 | 0 |
| Epithelioid sarcoma | 2 | 1 |
| Malignant fibrous histiocytoma | 6 | 1 |
| Synovial sarcoma | 6 | 0 |
| Ewing's tumour | 6 | 0 |
| Clear cell sarcoma | 2 | 0 |
| Rhabdomyosarcoma (embryonal) | 4 | 0 |
| Lelomyosarcoma | 6 | 1 |
| Osteosarcoma (soft tissue/bone) | 4 | 0 |

tumours of adipose differentiation and other benign and malignant soft tissue tumours, retrieved from the files of the Nuffield Orthopaedic Centre, were mounted onto glass slides and immunohistochemical screening with the aP2 antibody, used undiluted and diluted in phosphate-buffered saline (1:5, 1:10, 1:20, 1:30, 1:40, 1:50, 1:100), was carried out by a two-stage indirect immunoperoxidase technique as described previously.[7] Negative controls consisted of the addition of phosphate buffered saline instead of the primary antibody or the use of an irrelevant antibody of the same immunoglobulin subclass ($IgG_2$) [anticytokeratin CAM 5.2 (Becton-Dickinson Oxford UU)].

The number and range of tumours examined in this study are shown in Table 1. Histological criteria used for the identification of lipoblasts and for the pathological diagnosis of soft tissue tumours (such as malignant fibrous histiocytoma) examined in this study were those described in Enzinger and Weiss.[1] Where appropriate the various tumours were also stained immunohistochemically with monoclonal or polyclonal antibodies directed against specific cellular antigens known to be expressed by tumour cells in particular mesenchymal neoplasms (e.g. cytokeratin for synovial sarcoma and epithelioid sarcoma; MIC-2 for Ewing's sarcoma).

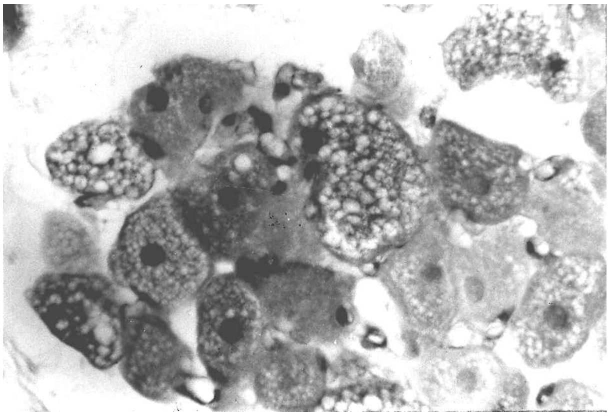

**Fig. 1.** *Indirect immunoperoxidase staining of a hibernoma showing staining of small fat vacuoles in brown fat cells for aP2 (1:30 dilution) (×400 original magnification).*

### Results aP2 protein expression, as identified by immunohistochemical staining, was noted in brown fat cells in hibernomas and lipoblasts in lipoblastomas and all types of liposarcoma (Table 1). Small fat vacuoles within the cytoplasm of brown fat cells in hibernomas reacted strongly for aP2 (Figure 1); the cell membrane was also stained but nuclei were unstained. Morphologically identifiable lipoblasts [i.e. univacuolated cells with a nucleus at one edge of the cell or multivacuolated cells with numerous lipid droplets in the central nucleus[1]], as well as spindle-shaped, round and pleomorphic cells, many of which contained small fat vacuoles, also stained for aP2 in well-differentiated, myxoid, round cell and pleomorphic liposarcomas (Figure 2).

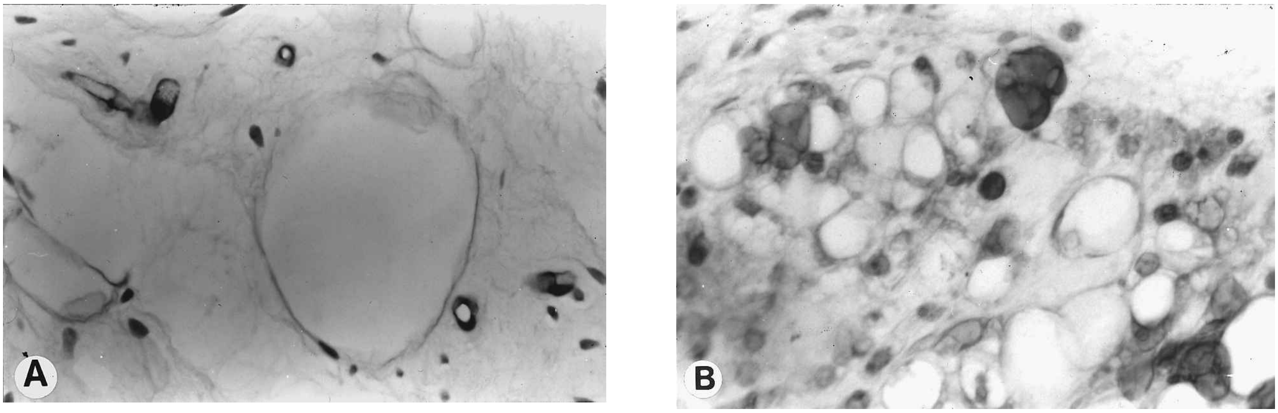

**Fig. 2.** *Indirect immunoperoxidase staining of (a) a myxoid liposarcoma and (b) a round cell liposarcoma showing positive aP2 staining of lipoblasts for aP2 (1:30 dilution) (×400 original magnification).*

There was marked variation in the extent of staining of lipoblasts and small fat cells within the various types of liposarcomas. To some degree this depended on the dilution of the primary antibody employed. Most lipoblasts stained when the antibody was used undiluted or when diluted at 1:5, 1:10 or 1:20; at these dilutions, however, antibody staining was not specific and other cell and tissue components, including endothelial cells and some mature fat cells, also reacted for aP2. More specific staining of lipoblasts was seen when the antibody was used at a relatively high dilution (1:30, 1:40 and 1:50). No staining of lipoblasts was seen when the antibody was used at 1:100 dilution. This pattern of staining was not affected by microwave or protease pre-treatment of sections. When used at a dilution of 1:30, 20–50% of lipoblasts in myxoid, round cell and pleomorphic liposarcomas reacted for aP2. At this dilution, fewer than 20% of lipoblasts reacted for aP2 in well-differentiated liposarcomas; however, as indicated above, the proportion of lipoblasts stained in this subtype of liposarcoma was much increased when the antibody was used at a lower dilution. It was noted that normal mature subcutaneous adipose tissue and mature fat cells in simple lipomas were negative for aP2 when the antibody was used at a high dilution (1:30 or more).

A single case of pleomorphic lipoma and two cases of spindle cell lipoma showed staining of small lipoblast-like fat cells for aP2 when the antibody was diluted more than 1:30. It was also found that isolated spindle-shaped tumour cells in some mesenchymal soft tissue tumours which were not of adipose differentiation, including cases of malignant fibrous histiocytoma (Figure 3), synovial sarcoma, leiomyosarcoma and myxoma, also reacted for aP2 when the antibody dilution was less than 1:30 (Table 1). No aP2 staining, however, was seen in cases of fibromatosis, benign fibrous histiocytoma, epithelioid sarcoma, Ewing's tumour, clear cell sarcoma, rhabdomyosarcoma, and osteosarcoma when the antibody was used at this dilution.

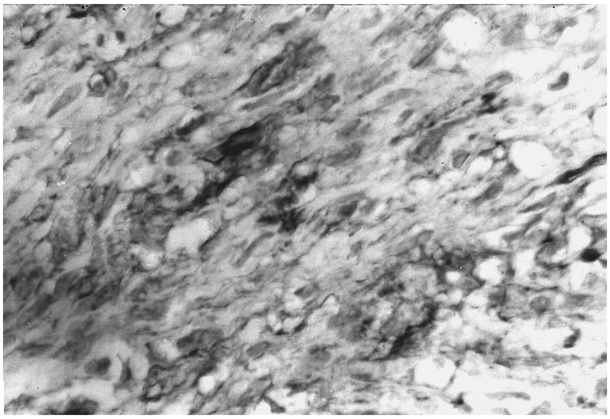

**Fig. 3.** *Indirect immunoperoxidase staining of a malignant fibrous histiocytoma showing positive aP2 staining of round and spindle-shaped tumour cells (1:10 dilution) (×250 original magnification).*

### Discussion

This study has confirmed that aP2 protein expression is found in lipoblasts and fetal fat cells in tumours of adipose differentiation, such as hibernoma, lipoblastoma and liposarcoma. aP2 was also expressed by lipoblast-like small fat cells in pleomorphic lipoma and spindle cell lipoma as well as by mesenchymal tumour cells in some non-adipose benign and malignant soft tissue tumours; this immunoreactivity was most marked when the antibody was used at an antibody dilution of 1:30 or less. These findings indicate that, although aP2 appears to be strongly expressed by lipoblasts and immature cells of the adipocyte lineage, this antigen is also expressed by some mature fat cells, endothelial cells and non-adipose mesenchymal tumour cells.

Genes regarded as early or late markers of adipocyte differentiation code for protein products which are associated with fat metabolism.[8,9] Although many of these proteins, including aP2, are mainly restricted to fat cells, their expression is generally not confined to cells of adipose differentiation.[10] Thus, our finding of aP2 expression in a few mesenchymal tumour cells which are not of the adipocyte lineage is not too surprising. The various types of mesenchymal cells which are found in connective tissue tumours are also thought to be derived from a common primordial stem cell and there may be conservation of this epitope by primitive cells present in these lesions.[11] In general, we found that mature adipose tissue and most benign adipose tissue tumours (with the exception of hibernoma and lipoblastoma) were largely negative for aP2 when the antibody identifying this antigen was used at a dilution of 1:30 or more. On this basis, aP2 expression is likely to be useful in the distinction of well-differentiated lipoma-like liposarcoma from simple lipoma. In general, lack of aP2 expression should also be useful in identifying small mature fat cells in benign adipose tissue tumours and small necrotic or degenerate fat cells in areas of fat necrosis or area of fibrosis in mature fat. Although all morphological variants of liposarcoma were found to stain for the aP2 protein, the extent of the immunohistochemical staining of lipoblasts was variable in terms of the number of cells stained and the strength of the reaction. aP2 staining, however, was noted in a few cases of spindle cell lipoma and pleomorphic lipoma, although the majority of these lesions were negative for aP2; although staining of these lesions may restrict the practical diagnostic use of this antibody, it should be noted that lipoblast-like cells have been identified ultrastructurally in both spindle cell lipoma and pleomorphic lipoma.[12,13,14]

aP2 expression is likely to be particularly valuable in tumour diagnosis as there are few specific histochemical markers of fat cell differentiation.[1,15,16] It should be particularly useful in distinguishing liposarcoma from other sarcomas containing numerous vacuolated pleomorphic/round tumour cells such as rhabdomyosarcoma, Ewing's sarcoma, clear cell sarcoma and leiomyosarcoma. It should also be useful in the differential diagnosis of myxoid tumours of soft tissue.[17] Lipoblasts in liposarcomas showed strong staining when the anti-aP2 antibody was used undiluted or diluted 1:5. 1:10 or 1.20; however, it was found that use of this antibody at this dilution resulted in some loss of specificity with cells other than lipoblasts being stained. More specific staining was seen when the antibody was used at a dilution greater than 1:30. As aP2 expression in malignant tumours is generally restricted to liposarcoma when the monoclonal antibody is used at a dilution of 1:20 or more our results suggest that at this dilution the anti-aP2 antibody is best employed as part of an antibody panel to characterise the immunophenotype of tumour cells in malignant soft tissue neoplasms.

### Acknowledgements

The authors would like to thank Mrs C Costar for typing the manuscript. This study was supported by the British Orthopaedic Association Wishbone Trust.

### References

1 Enzinger FM, Weiss SW. Soft tissue tumours. St Louis: Mosby, 1995.

2 Bennett JH, Shousha S, Puddle B, Athanasou NA. Immunohistochemical identification of tumours of adipocytic differentiation using an antibody to aP2 protein. *J Clin Pathol* 1995; 48:950–954.

3 Joyner CJ, Triffitt JT, Puddle B, Athanasou NA. Development of a monoclonal antibody to the aP2 protein to identify adipocyte precursors in tumours of adipose differentiation. *Pathol. Res Pract* 1999; 195:461–466.

4 Bernlohr DA, Angus CW, Lane MD, Bolanowski MA, Kelly T. Expression of specific mRNAs during adipose differentiation, identification of an mRNA encoding a homologue of myelin P2 protein. *Proc Natl Acad Sci USA* 1984; 81:5468–5472.

5 Bernlohr DA, Doering TL, Kelly TJ, Lane MD. Tissue specific expression of p422 protein, a putative lipid carrier, in mouse adipocytes. *Biochem Biophys Res Commun* 1985; 132:850–855.

6 Baxa CA, Sha RS, Buelt MK, Smith AJ, Smith AJ, Matarese V, Chinander LL, *et al.* Human adipocyte lipid-binding protein: purification of the protein and cloning of its complementary DNA. *Biochemistry* 1989; 28:8683–8690.

7 Sternberger A. Immunocytochemistry. New York: Wiley, 1986.

8 Poissonet CM, La Velle M, Burds AR. Growth and development of adipose tissue. *J Pediatr* 1988; 113:1–9.

9 Spiegelman BM, Choy L, Hotamisligil GS, Graves RA, Tontonoz P. Regulation of adipocyte gene expression in differentiation and syndromes of obesity/diabetes. *J Biol Chem* 1993; 268:6823–6926.

10 Ailhaud G, Amri E, Barbaras R, Casteilla L, Catolioto RM, Dani C, *et al.* Adipose cell differentiation: differential expression of specific mRNAs and protein markers in cells from white and brown adipose tissues. In: Berry EM, Blondheim SH, Eliahou HE, Shafir E, eds. Recent advances in obesity research. Vol V. London, Paris: John Libbey, 1987:174–180.

11 Beresford JN, Bennett JH, Delvin C, Le Boys PS, Owen ME. Evidence for an inverse relationship between the differentiation of adipocyte and osteogenic cells in rat marrow stromal cell cultures. *J Cell Science* 1992; 102:341–351.

12 Shmookler BM, Enzinger FM. Pleomorphic lipoma: a benign tumour simulating liposarcoma. A clinicopathological analysis of 48 cases. *Cancer* 1981; 47:126–133.

13 Azzopardi JG, Iocco J, Salm R. Pleomorphic lipoma: a tumour simulating liposarcoma. *Histopathology* 1983; 7:511–423.

14 Dupree WB Progress in pseudosarcomatous and borderline soft tissue tumours. In: Fletcher CD, McKee PH (eds) Pathobiology of soft tissue tumours. Edinburgh, Churchill Livingstone 1990; 256–294.

15 Mackenzie DH, Filipe MI. Soft tissue tumours. In: Filipe MI, Lake BD (eds) Histochemistry in Pathology, Churchill Livingstone 1983; 245–251.

16 Kilpatrick SE, Doyon J, Choong PFM, Sim FH, Nascimento AG. (1996). The clinicopathological spectrum of myxoid and round cell liposarcoma. A study of 95 cases. *Cancer* 77:1450–1458.

17 Graadt van Roggen JF, Hogendoorn PCW, Fletcher CDM. (1999). Myxoid tumours of soft tissue. *Histopathology* 35:291–312.
